# Comparative genomic analysis provides insight into the phylogeny and virulence of atypical enteropathogenic *Escherichia coli* strains from Brazil

**DOI:** 10.1371/journal.pntd.0008373

**Published:** 2020-06-01

**Authors:** Rodrigo T. Hernandes, Tracy H. Hazen, Luís F. dos Santos, Taylor K. S. Richter, Jane M. Michalski, David A. Rasko

**Affiliations:** 1 Departamento de Microbiologia e Imunologia, Instituto de Biociências, Universidade Estadual Paulista “Júlio de Mesquita Filho” (UNESP), Botucatu, SP, Brasil; 2 Institute for Genome Sciences, Department of Microbiology and Immunology, University of Maryland School of Medicine, Baltimore, Maryland, United States of America; 3 Instituto Adolfo Lutz, São Paulo, SP, Brasil; Beijing Institute of Microbiology and Epidemiology, CHINA

## Abstract

**Background:**

Atypical enteropathogenic *Escherichia coli* (aEPEC) are one of the most frequent intestinal *E*. *coli* pathotypes isolated from diarrheal patients in Brazil. Isolates of aEPEC contain the locus of enterocyte effacement, but lack the genes of the bundle-forming pilus of typical EPEC, and the Shiga toxin of enterohemorrhagic *E*. *coli* (EHEC). The objective of this study was to evaluate the phylogeny and the gene content of Brazilian aEPEC genomes compared to a global aEPEC collection.

**Methodology:**

Single nucleotide polymorphism (SNP)-based phylogenomic analysis was used to compare 106 sequenced Brazilian aEPEC with 221 aEPEC obtained from other geographic origins. Additionally, Large-Scale BLAST Score Ratio was used to determine the shared versus unique gene content of the aEPEC studied.

**Principal Findings:**

Phylogenomic analysis demonstrated the 106 Brazilian aEPEC were present in phylogroups B1 (47.2%, 50/106), B2 (23.6%, 25/106), A (22.6%, 24/106), and E (6.6%, 7/106). Identification of EPEC and EHEC phylogenomic lineages demonstrated that 42.5% (45/106) of the Brazilian aEPEC were in four of the previously defined lineages: EPEC10 (17.9%, 19/106), EPEC9 (10.4%, 11/106), EHEC2 (7.5%, 8/106) and EPEC7 (6.6%, 7/106). Interestingly, an additional 28.3% (30/106) of the Brazilian aEPEC were identified in five novel lineages: EPEC11 (14.2%, 15/106), EPEC12 (4.7%, 5/106), EPEC13 (1.9%, 2/106), EPEC14 (5.7%, 6/106) and EPEC15 (1.9%, 2/106). We identified 246 genes that were more frequent among the aEPEC isolates from Brazil compared to the global aEPEC collection, including *espG2*, *espT* and *espC* (*P*<0.001). Moreover, the *nleF* gene was more frequently identified among Brazilian aEPEC isolates obtained from diarrheagenic patients when compared to healthy subjects (69.7% vs 41.2%, *P*<0.05).

**Conclusion:**

The current study demonstrates significant genomic diversity among aEPEC from Brazil, with the identification of Brazilian aEPEC isolates to five novel EPEC lineages. The greater prevalence of some virulence genes among Brazilian aEPEC genomes could be important to the specific virulence strategies used by aEPEC in Brazil to cause diarrheal disease.

## Author summary

Atypical EPEC (aEPEC) is one of the most frequent diarrheagenic *Escherichia coli* pathotypes isolated from patients in Brazil and is associated with diarrheal outbreaks. This study is the first to sequence the genomes of a collection of aEPEC isolates from a South American country, Brazil, and compare their phylogenetic relationships and gene content with a global collection of aEPEC. This approach identified Brazilian aEPEC genomes in previously characterized EPEC/EHEC phylogenomic lineages and resulted in the identification of five novel EPEC phylogenomic lineages, designated EPEC11 to EPEC15. We also observed that virulence genes, such as *espG2*, *espT* and *espC* were more frequently identified among the Brazilian aEPEC genomes, demonstrating potential differences in the virulence repertoire of this pathogen in Brazil.

## Introduction

The enteropathogenic *Escherichia coli* (EPEC) pathotype is divided into two subgroups: typical EPEC (tEPEC) and atypical EPEC (aEPEC) [1]. The main difference between these two groups is the presence of the EPEC adherence factor plasmid (pEAF) encoding the bundle forming pilus (BFP) in tEPEC, and its absence in aEPEC [1, 2]. A previous epidemiological study demonstrated that tEPEC was a significant cause of moderate to severe diarrhea and increased mortality in infants [3]. In recent decades aEPEC has emerged as a frequent cause of diarrheal disease in children and adults, and has been linked to several diarrheal outbreaks [2, 4–12]. In particular, aEPEC isolates of serotypes O2:H16, O33:H34, O39:H9, O108:H^-^ and ONT:H19 were implicated in five distinct diarrheal outbreaks in São Paulo State, Brazil, in 2013 [8].

The main virulence feature of EPEC is its ability to produce the histopathological attaching and effacing (A/E) lesions on the surface of infected host cells [13]. The A/E lesion is characterized by the destruction of the microvilli brush border, intimate bacteria-cell adherence and formation of pedestal-like structures rich in F-actin and other cytoskeleton elements, which causes intestinal epithelial cells to have a reduced absorptive capacity [13, 14]. The 41 genes associated with this phenotype are located in a pathogenicity island (PAI) termed the locus of enterocyte effacement (LEE) [15]. The LEE region encodes all the structural components of the type 3 secretion system (T3SS), as well as seven effector proteins, the intimin outer membrane protein, the intimin translocated receptor protein (Tir), chaperones, and transcriptional regulators [15–18]. Although enterohemorrhagic *E*. *coli* (EHEC) similarly contain the LEE and produce the A/E lesion on infected epithelial cells, EHEC also contain the *stx* genes, encoding the Shiga toxin, which is not present in EPEC [13]. Besides the T3SS effectors located in the LEE region, effectors encoded outside of LEE, known as non-LEE encoded effectors, have emerged as important accessory virulence factors in A/E pathogens [19, 20]. These effectors are responsible for inducing several modifications in the host cells during infection, including inhibition of apoptosis, interference with inflammatory signaling pathways, invasion, tight junction disruption and phagocytosis [19, 20].

Previous comparative genomic studies have demonstrated that EPEC exhibit considerable genomic diversity, with representatives in at least 10 different phylogenomic lineages [21, 22]. This is most likely due to the acquisition of the LEE region and its stable retention by genomically diverse lineages of *E*. *coli* [23]. These prior studies have focused on investigating the genomic diversity of EPEC isolates from locations in Europe, Africa, and Asia [22, 23]. While aEPEC poses a significant diarrheal burden in South America [2, 9, 24], to our knowledge there have been no previous comparative genomics studies that have investigated the genetic diversity of aEPEC from Brazil or other countries in South America. Thus, the goal of this study was to analyze the genetic relatedness and virulence gene content of Brazilian aEPEC isolates compared to a globally distributed collection of aEPEC.

## Methods

### DNA extraction, genome sequencing, and assembly

Genomic DNA of the 106 aEPEC isolates from Brazil (S1 Table) was extracted from overnight cultures using the Sigma GenElute bacterial genomic DNA kit (Sigma-Aldrich; St. Louis, MO). The genomes were sequenced using paired-end libraries on the Illumina HiSeq 4000. The 150 bp Illumina reads were assembled using SPAdes v.3.7.1 [25], and the final assemblies were filtered to contain contigs that were ≥500 bp in length and had ≥5X kmer coverage.

### Phylogenomic analysis

A single nucleotide polymorphism (SNP)-based phylogenomic analysis was performed on the 106 Brazilian aEPEC (S1 Table), 221 global aEPEC publicly available from previous studies [22, 23] (S2 Table, S1 Fig), and a collection of 48 diverse *E*. *coli* and *Shigella* genomes (S3 Table). A total of 179,202 SNPs were detected relative to the completed genome sequence of the phylogroup F laboratory isolate *E*. *coli* IAI39 (accession number: NC_011750) using the *In Silico* Genotyper (ISG) [26]. A maximum-likelihood phylogeny with 100 bootstrap replicates was generated using RAxML v.7.2.8 [27] with the GTR model of nucleotide substitution, the GAMMA model of rate heterogeneity, and visualized using FigTree v.1.4.2 (http://tree.bio.ed.ac.uk/software/figtree/).

### *In silico* Multilocus Sequence Typing (MLST) and serotyping

The seven loci of the multilocus sequence typing (MLST) scheme (*adk*, *gyrB*, *fumC*, *icd*, *mdh*, *purA*, and *recA*) developed by Wirth et al. [28] were identified in each of the genomes listed in S1 and S2 Tables using BLASTN, and MLST sequence type (ST) were determined by querying the PubMLST database (https://pubmlst.org).

The molecular serotype of each genome analyzed was determined using Serotype Finder 1.1 (https://cge.cbs.dtu.dk/services/SerotypeFinder/) with the default settings of an 85% identity threshold and 60% minimum alignment length [29].

### Large-Scale BLAST Score Ratio (LS-BSR) analysis

The 106 Brazilian aEPEC and additional A/E *E*. *coli* genomes (S1 to S3 Tables) analyzed in this study were compared by BLASTN LS-BSR analysis as previously described [30]. Briefly, the predicted protein-encoding genes of each genome that had ≥90% nucleotide sequence identity and ≥90% alignment length were assigned to gene clusters using CD-HIT v.4.6.4 [31] (S1 Data Set). The predicted protein function of each gene cluster was determined with an ergatis-based [32] in-house annotation pipeline [33]. The presence of gene clusters with significant similarity in each of the five novel phylogenomic lineages identified in this study (EPEC11, EPEC12, EPEC13, EPEC14 and EPEC15) were defined by using an LS-BSR value ≥0.9; while the absence in the remaining A/E genomes were defined by using an LS-BSR value ≤0.4.

Additionally, we examined the occurrence of genes that were more common among the Brazilian aEPEC isolates when compared to the isolates from outside of Brazil, as well as among Brazilian and global aEPEC identified in the same phylogenomic lineage. The presence of each gene cluster was defined as having a BLASTN LS-BSR value ≥0.8, and differences in genes frequency among the two group of aEPEC studied (Brazilian versus Global) were tested by using the Chi-square test with Yates correlation and two-tailed Fisher's exact test.

### *In silico* detection of virulence factor-encoding genes and intimin subtyping

TBLASTN LS-BSR was used to detect the presence of known EPEC virulence factor-encoding genes in the 106 Brazilian aEPEC genomes, as previously described [22, 34]. The virulence factor-encoding genes investigated included the repertoire of Non-LEE effectors present in the typical EPEC prototypes E2348/69 [35] and B171 [36]; since many of them has been associated with the EPEC pathogenesis [20], as well as *espT* [37] and *espV* [38], first indentified and functionally characterized in the outbreak-associated aEPEC E110019. In addition, we searched for genes reponsible for encoding adhesins (*efa1*/*lifA*, *iha*, *paa*, *toxB*, *cah*, *ehaA*, *ehaB*, *ehaC*, *ehaD*, *ehaG*, and *ehaJ*) and toxins (*astA*, *pic*, *pet ehxA*, *espP*, *sepA*, *sigA*, and *espC*) that haven been frequentelly deteceded among aEPEC isolates, demontrated to contribute to aEPEC pathogenesis and/or previously associated with diarrheal disease [8, 39–42]. The intimin subtypes [43, 44] were identified in the 106 aEPEC genomes analyzed using BLASTN LS-BSR as described above.

## Results

### Characteristics of the Brazilian aEPEC genomes

A total of 106 aEPEC isolates obtained from previous studies performed in Brazil [5, 8, 9, 45] were sequenced. This collection of Brazilian aEPEC were isolated over a span of seven years from 2009 to 2016, and were associated with multiple clinical presentations, including 17 isolates from subjects with asymptomatic infection, 76 from patients with diarrhea, and 13 from diarrheal patients investigated in five distinct diarrheal outbreaks (S1 Table). The average genome size of the Brazilian aEPEC genome assemblies was 4,927,030 bp (range 4,551,111 to 5,442,868), and the average GC content was 50.53% (range: 50.13% to 50.78%), which is consistent with previous studies of sequenced *E*. *coli* genomes [22, 46] (S1 Table).

### The majority of the aEPEC Brazilian genomes were identified in phylogroup B1

**Fig 1:**
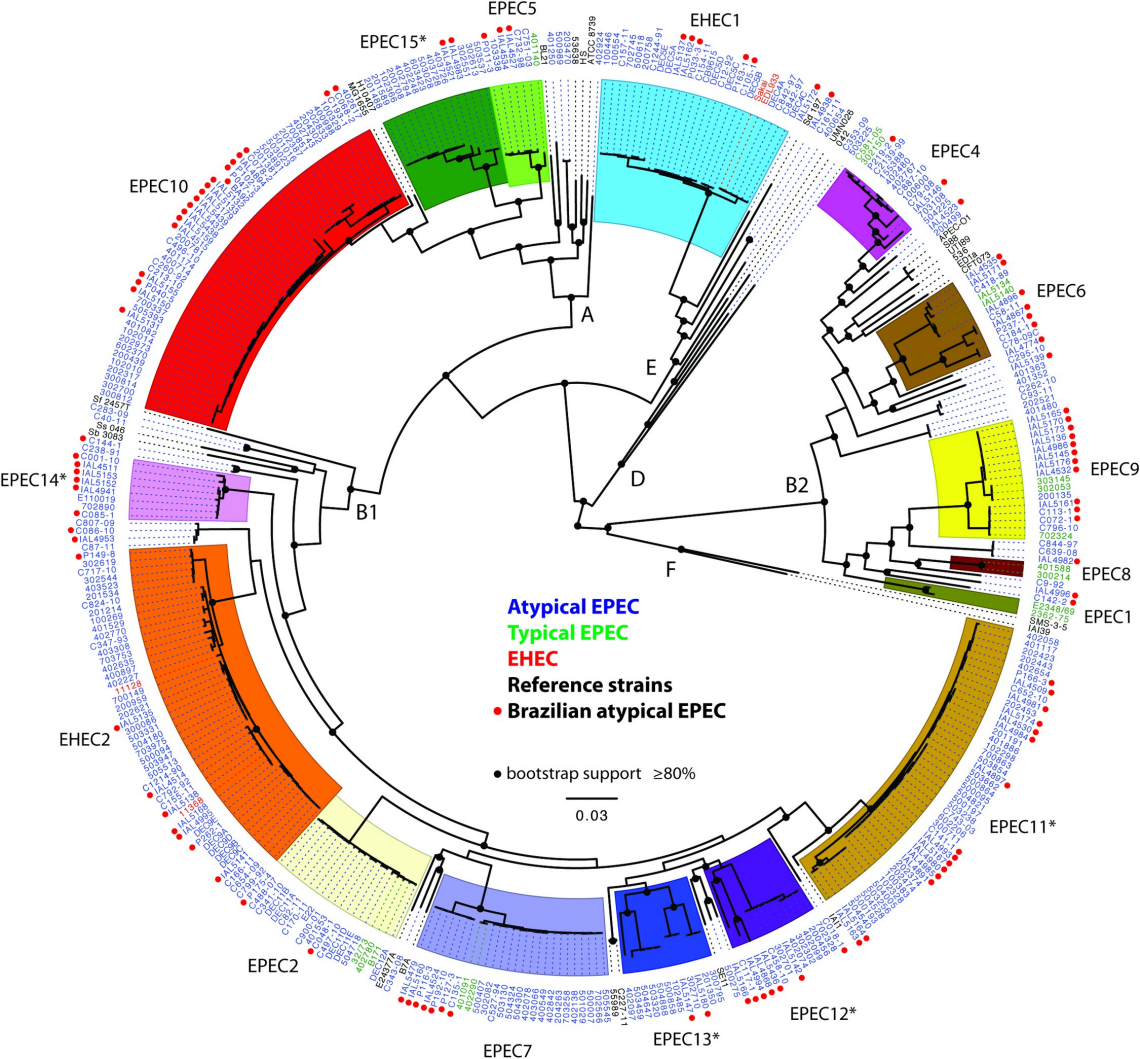
Phylogenomic analysis of aEPEC from Brazil compared to aEPEC from other geographic origins. Phylogenomic analysis of atypical EPEC genomes (labeled in blue), compared to typical EPEC (labeled in green), EHEC (labeled in red) and reference *E*. *coli* and *Shigella* genomes (labeled in black). Red circles indicate the 106 Brazilian aEPEC genomes. The clades in the phylogeny are colored by phylogenomic lineage, and the *E*. *coli* phylogroups are indicated by the letters: A, B1, B2, D, E, and F. An asterisk (*) indicates the five novel EPEC phylogenomic lineages identified in this study. The nodes with bootstrap values >80% are labeled with a black circle.

The 106 Brazilian aEPEC genomes were identified in phylogroup B1 (47.2%, 50/106), followed by phylogroups B2 (23.6%, 25/106), A (22.6%, 24/106), and E (6.6%, 7/106) (Fig 1, Table 1). The 221 global aEPEC genomes analyzed had a similar phylogroup distribution with phylogroup B1 (55.7%, 123/221) being the most common, followed by phylogroups A (22.6%, 50/221) and B2 (11.3%, 25/221), while the aEPEC genomes of phylogroups E (9.5%, 21/221) and D (0.9%, 2/221) were the least common (Table 1). While the phylogroup distributions of Brazilian aEPEC were similar to those of the global aEPEC genomes, there were significantly more phylogroup B2 genomes identified among the Brazilian aEPEC (23.6%, 25/106) compared with the global collection (11.3%, 25/221) (Table 1) (*P*<0.05). The reasons for a greater prevalence of phylogroup B2 aEPEC in Brazil compared with the global collections are not clear at this time and may be representative of an isolation or sequencing bias.

**Table 1:** Classification of the aEPEC genomes in the distinct phylogroups and phylogenomic lineages.

Phylogroup/ Phylogenomic lineage	No. (%) of atypical EPEC (aEPEC)			*P* value
	Brazil (*n* = 106)	Global (*n* = 221)	Total (*n* = 327)	
Phylogroup A	**24 (22.6)**	**50 (22.6)**	**74 (22.6)**	0.9972^b^
**EPEC5**	3 (2.8)	3 (1.4)	6 (1.8)	0.3937^c^
**EPEC10**	19 (17.9)	30 (13.6)	49 (15.0)	0.3865^b^
**EPEC15**	2 (1.9)	13 (5.9)	15 (4.6)	0.157^c^
**uEPEC**^**a**^	0	4 (1.8)	4 (1.2)	0.3086^c^
Phylogroup B1	**50 (47.2)**	**123 (55.7)**	**173 (52.9)**	0.1866^b^
**EHEC2**	8 (7.5)	37 (16.7)	45 (13.8)	0.0368^b^
**EPEC2**	2 (1.9)	13 (5.9)	15 (4.6)	0.1570^c^
**EPEC7**	7 (6.6)	17 (7.7)	24 (7.3)	0.8991^b^
**EPEC11**	15 (14.2)	30 (13.6)	45 (13.8)	0.8874^b^
**EPEC12**	5 (4.7)	7 (3.2)	12 (3.7)	0.5348^c^
**EPEC13**	2 (1.9)	10 (4.5)	12 (3.7)	0.3499^c^
**EPEC14**	6 (5.7)	2 (0.9)	8 (2.4)	0.0159^c^
**uEPEC**^**a**^	5 (4.7)	7 (3.2)	12 (3.7)	0.5348^c^
Phylogroup B2	**25 (23.6)**	**25 (11.3)**	**50 (15.3)**	0.0065^b^
**EPEC4**	2 (1.9)	8 (3.6)	10 (3.1)	0.5090^c^
**EPEC6**	6 (5.7)	3 (1.4)	9 (2.8)	0.0629^c^
**EPEC9**	11 (10.4)	2 (0.9)	13 (4.0)	0.0001^c^
**uEPEC**^**a**^	6 (5.7)	12 (5.4)	18 (5.5)	0.9318^b^
Phylogroup D	**0**	**2 (0.9)**	**2 (0.6)**	1.0000^c^
**uEPEC**^**a**^	0	2 (0.9)	2 (0.6)	1.0000^c^
Phylogroup E	**7 (6.6)**	**21 (9.5)**	**28 (8.6)**	0.5056^b^
**EHEC1**	5 (4.7)	19 (8.6)	24 (7.3)	0.3017^b^
**uEPEC**^**a**^	2 (1.9)	2 (0.9)	4 (1.2)	0.5978^c^

^a^Unclassified EPEC (uEPEC) refer to atypical EPEC genomes not classified in any of the pre-existing EPEC/EHEC phylogenomic lineages or in the five novel EPEC lineages described in this study.

^b,c^Differences observed between aEPEC genomes from Brazil and from a global distribution were determined using Chi-square test with Yates correlation (^b^) or two-tailed Fisher's exact test (^c^).

### Brazilian aEPEC genomes were identified in previously described EPEC/EHEC phylogenomic lineages

Previous genetic studies focusing on understanding the evolution of EPEC have demonstrated that isolates from this pathotype can belong to at least ten distinct EPEC phylogenomic lineages, which have been designated EPEC1 to EPEC10 [21, 47, 48], in addition to unclassified EPEC isolates, that exist outside of these defined EPEC lineages. We observed that 59.4% (63/106) of the aEPEC Brazilian genomes could be classified into previously defined phylogenomic lineages: EPEC2 (1.9%, 2/106), EPEC4 (1.9%, 2/106), EPEC5 (2.8%, 3/106), EPEC6 (5.7%, 6/106), EPEC7 (6.6%, 7/106), EPEC9 (10.4%, 11/106), EPEC10 (17.9%, 19/106), as well as a small proportion in EHEC lineages, EHEC1 (4.7%, 5/106) and EHEC2 (7.5%, 8/106) (Fig 1, Table 1).

The greatest number of Brazilian aEPEC genomes were identified in the EPEC10 lineage (17.9%, 19/106). The majority of the Brazilian aEPEC in this lineage were MLST ST10 (78.9%, 15/19), including seven aEPEC isolates with serotype O2:H16 (36.8%, 7/19). We also observed a significantly greater number of Brazilian aEPEC genomes than global aEPEC (10.4% vs 0.9%, *P*<0.001) in the EPEC9 lineage (Table 1). In this lineage we observed eight Brazilian aEPEC of serotype O33:H34 (72.7%, 8/11). Of note, the Brazilian aEPEC genomes identified with serotypes (sequence types) O2:H16 (ST10) and O33:H34 (ST1951), were obtained from diarrheal outbreaks that occurred in Brazil in 2013 (4 isolates O2:H16 and 4 isolates O33:H34, each serotype from a distinct outbreak), as well as from sporadic cases of diarrhea that occurred in Brazil over several years (3 isolates O2:H16 and 4 isolates O33:H34) (S1 Table), while in the global aEPEC collection, only one isolate, from Mali (isolate 200135), was identified with serotype O33:H34 (ST2346); and aEPEC of serotype O2:H16 were not detected (S2 Table). The differences observed in the frequency of aEPEC of serotypes O2:H16 (6.6% vs 0.0%, *P*<0.001) and O33:H34 (7.5% vs 0.5%, *P*<0.001), among the Brazilian vs Global aEPEC collections, may be due to these serotypes being associated with Brazilian diarrheal outbreaks.

Brazilian aEPEC identified in the lineages EHEC1 and EHEC2 had the serotypes O55:H7 (100.0%, 5/5) and O26:H11 (75.0%, 6/8), respectively. The five O55:H7 were ST335 (Table 2) and were most closely related to the O157:H7 of the EHEC1 lineage (Fig 1). The six aEPEC with serotype O26:H11 in the EHEC2 lineage were one of two MLST sequence types: ST21 (50.0%, 3/6) or ST29 (50.0%, 3/6) (Table 2). The number of aEPEC genomes identified in the EHEC2 lineage was significantly higher among the global collection of aEPEC than the Brazilian aEPEC (7.5% vs 16.7%, *P*<0.05) (Table 1).

**Table 2:** Characteristics of the 106 Brazilian aEPEC genomes analyzed in this study.

Phylogroup	Phylogenomic Lineage	MLST ST	Intimin Subtype	*In Silico* Serotype (No. of aEPEC isolates)
**A (22.6%)**	EPEC5 (2.8%)	206	kappa	O88:H5 (2), O118/O151:H5 (1)
	EPEC10 (17.9%)	10	beta	ONT:H16 (7)^a^
			epsilon	O157:H16 (3)
			omicron	O13/O135:H11 (1)
			theta	O51:H40 (1), O117:H40 (3)
		34	lambda	O101:H33 (2)
		ND	theta	O127:H40 (2)
	EPEC15 (1.9%)	301	omicron	O76:H2 (1)
			xi	O80:H2 (1)
**B1 (47.2%)**	EHEC2 (7.5%)	21	beta	O26:H11 (3)
		29	beta	O26:H11 (3)
			theta	ONT:H9 (1)
		4550	beta	O103:H8 (1)
	EPEC2 (1.9%)	20	beta	O119:H2 (1), O128ab/ac:H2 (1)
	EPEC7 (6.6%)	642	omicron	O85:H4 (3), O103:H4 (2), O184:H4 (1), ONT:H4 (1)
	EPEC11 (14.2%)	517	epsilon2	O35:H19 (1), O71:H19 (1), O88:H25 (2), O123/O186:H19 (3), O126:H19 (3), ONT:H19 (1)
			eta	O160:H19 (2)
		5241	eta	O108:HNT (2)
	EPEC12 (4.7%)	40	theta	O66:H21 (1)
			beta	O109:H21 (1)
		101	eta	O21:H21 (1)
		224	epsilon	O182:H23 (1)
		ND	eta	O91:H23 (1)
	EPEC13 (1.9%)	337	theta	O108:H21 (1)
		442	theta	O146:H21 (1)
	EPEC14 (5.7%)	381	iota	O39:H9 (2)
			zeta	O177:H9 (1), ONT:H12 (1)
		590	iota	O98:H8 (1), ONT:H8 (1)
	uEPEC (4.7%)	300	zeta	O182:H25 (1)
		327	theta	O111:H8 (1), O115:H8 (1)
		2178	theta	O170:H49 (1)
		2338	theta	O131:H46 (1)
**B2 (23.6%)**	EPEC4 (1.9%)	4601	beta2	ONT:H6 (1)
		6323	beta2	O56:H6 (1)
	EPEC6 (5.7%)	122	alpha2	O63:H6 (1)
		526	iota	O145:H34 (1)
		582	alpha2	O132:H34 (1)
		583	alpha2	O63:H6 (2)
		713	iota	O145:H34 (1)
	EPEC9 (10.4%)	1951	lambda	O33:H34 (8)
		2346	alpha	O142:H34 (3)
	uEPEC (5.7%)	941	zeta	O156:H1 (1)
		1040	alpha	O177:H45 (1)
		2201	mu	O63:H34 (1)
		5428	kappa	O71:H49 (1)
		5575	alpha2	O125ac:H49 (1)
		ND	alpha	O37:H45 (1)
**E (6.6%)**	EHEC1 (4.7%)	335	gamma	O55:H7 (5)
	uEPEC (1.9%)	32	gamma	O145:HNT (1)
		2569	theta	ONT:H31 (1)

^a^Previously defined as serotype O2:H16 by standard agglutination tests with absorbed antisera (9, 45).

### Designation of five novel EPEC phylogenomic lineages

Phylogenomic analysis of Brazilian aEPEC genomes along with aEPEC genomes from a global distribution, led to the identification of five novel phylogenomic lineages with five or more genomes in each, which we have designated EPEC11 to EPEC15 (Fig 1). The EPEC11 to EPEC14 lineages were identified in phylogroup B1, whereas EPEC15 was identified in phylogroup A (Fig 1). Among the aEPEC Brazilian genomes analyzed, we observed that 28.3% (30/106) were identified in one of the five novel EPEC lineages. The distribution of these isolates is as follow: EPEC11 (14.2%, 15/106), EPEC12 (4.7%, 5/106), EPEC13 (1.9%, 2/106), EPEC14 (5.7%, 6/106) and EPEC15 (1.9%, 2/106) (Table 1). It is important to highlight the number of Brazilian aEPEC genomes identified in the EPEC11 lineage (14.2%, 15/106) (Table 1), which are second in abundance after the Brazilian aEPEC isolates identified in the EPEC10 lineage (17.9%, 19/106) (Table 1). The Brazilian aEPEC identified in EPEC11 had eight distinct serotypes (O35:H19, O71:H19, O88:H25, O108:HNT, O123/O186:H19, O126:H19, O160:H19 and ONT:H19), harbored intimin subtype epsilon2 (73.3%, 11/15) or eta (26.7%, 4/15), and the majority (86.7%, 13/15) were ST517 (Tables 2 and 3). Moreover, we observed that the aEPEC archetype isolate E110019, from an outbreak that occurred in Finland [49], was present in the newly designated EPEC14 lineage (Fig 1), along with two Brazilian aEPEC isolates (IAL5152 and IAL 5153) which were serotype O39:H9 and obtained from an outbreak (S1 Table). It is important to emphasize that the number of Brazilian aEPEC genomes identified in EPEC14, a novel EPEC lineage, is statistically different when comparing with aEPEC from outside of Brazil (5.7% vs 0.9%, *P*<0.05) (Table 1). Additionally, 12.3% (13/106) of the Brazilian aEPEC genomes identified in phylogroups B1 (5 isolates), B2 (6 isolates) and E (2 isolates) were not classified into any of the described EPEC or EHEC phylogenomic lineages (Tables 1 and 2).

**Table 3:** Serotypes and Multilocus sequence typing (MLST) of the aEPEC isolates classified in the five novel phylogenomic lineages of EPEC described in this study.

Phylogroup	Phylogenomic Lineage	MLST ST	aEPEC *in silico* Serotype:	
			From Brazil	From other origins
B1	EPEC11 (*n* = 45)	517	O35:H19 (1) O71:H19 (1) O88:H25 (2) O123/O186:H19 (3) O126:H19 (3) O160:H19 (2) ONT:H19 (1)	O71:H19 (6) O88:H25 (1) O111:H19 (4) O116:H9 (3) O123/O186:H19 (3) O136:H51 (1) O160:H19 (2) O165:H9 (2) O171:H19 (1) ONT:H19 (1)
		5241	O108:HNT (2)	O8:H19 (5)
		5485	-	O171:H19 (1)
	EPEC12 (*n* = 12)	40	O66:H21 (1) O109:H21 (1)	O109:H21 (6) ONT:H21 (1)
		101	O21:H21 (1)	-
		224	O182:H23 (1)	-
		ND	O91:H23 (1)	-
	EPEC13 (*n* = 12)	337	O108:H21 (1)	O108:H21 (2)
		442	O146:H21 (1)	O146:H21 (2)
		443	-	O64:H21 (4) O161:H21 (1)
		5508	-	O104:H7 (1)
	EPEC14 (*n* = 8)	381	O39:H9 (2) O177:H9 (1) ONT:H12 (1)	O177:H9 (1) ONT:H9 (1)
		590	O98:H8 (1) ONT:H8 (1)	-
A	EPEC15 (*n* = 15)	301	O76:H2 (1) O80:H2 (1)	O45:H19 (6) O61:HNT (1) O80:H2 (2)
		382	-	ONT:H5 (3)
		4590	-	ONT:H2 (1)

### Identification of genes that are unique among the novel EPEC phylogenomic lineages

We used LS-BSR to identify gene content that is present in one group of isolates and lacking in another. First, we examined the existence of genes that were present in each of the five novel EPEC lineages (EPEC11, EPEC12, EPEC13, EPEC14 and EPEC15) and absent in all other A/E *E*. *coli* genomes included in this study. A total of 936 gene clusters were found exclusively, at a variable frequency, in the five novel EPEC phylogenomic lineages as follows: 196 (EPEC11), 181 (EPEC12), 241 (EPEC13), 140 (EPEC14), and 178 gene clusters (EPEC15) (Table 4). However, only one of these gene clusters was identified in all of the genomes within its associated lineage and absent from all other genomes analyzed. The EPEC11 lineage harbors a single gene cluster (termed CYCE01000006.1_37), that encodes a hypothetical protein, present in all 45 (100.0%) aEPEC genomes from this phylogenomic lineage and absent in all other A/E genomes analyzed (Table 4). A summary of the LS-BSR analyses can be found in Table 4, and all genes exclusively present in each of the five novel phylogenomic lineages are listed in S4 Table.

**Table 4:** Detection of gene clusters that are unique to each of the novel EPEC phylogenomic lineages.

Phylogenomic Lineages (number of genomes)^c^	No. of gene clusters exclusively present in each phylogenomic lineage^a^^,^^b^:				
	100% of the Genomes	≥90% of the Genomes	≥75% of the Genomes	≥50% of the Genomes	≥1 of the Genomes
EPEC11 (*n* = 45)	1	2	2	2	196
EPEC12 (*n* = 12)	0	0	2	11	181
EPEC13 (*n* = 12)	0	0	0	0	241
EPEC14 (*n* = 8)	0	0	3	13	140
EPEC15 (*n* = 15)	0	0	0	12	178

^a^Total gene clusters: 24,383

^b^The presence of gene clusters in each of the five novel phylogenomic lineages was defined using a LS-BSR value ≥0.9, and absence in the remaining A/E genomes analyzed were defined by a LS-BSR value ≤0.4. The gene clusters detected exclusively in each of the five novel phylogenomic lineage are listed in S4 Table.

^c^The number of attaching and effacing (typical EPEC, atypical EPEC and EHEC) genomes analyzed was 349, as described in S1, S2 and S3 Tables.

### Differential prevalence of gene content between Brazilian aEPEC compared to a global collection of aEPEC

Since so few genes were exclusively present or absent in the novel phylogenomic lineages we wanted to examine if there were any genes that were more statistically prevalent among the Brazilian aEPEC isolates when compared to the isolates obtained from locations outside of Brazil. This analysis of the gene content led to the identification of 246 genes that were statistically more frequent (*P*<0.001) among the Brazilian aEPEC genomes than aEPEC from other geographic origins (Brazilian aEPEC vs Global aEPEC). Included among these genes were two T3SS-effectors: *espG2*, gene cluster 128_11_7_153 (20.8% vs 6.8%), and *espT*, gene clusters 148_12_48_29 (21.7% vs 3.6%) and CYEQ01000057.1_8 (17.9% vs 3.2%). The *espC* gene, encodes an autotransporter protein [50], represented by gene clusters 1024_15_1_31 (27.4% vs 7.2%) and 276_12_73_9 (26.4% vs 8.1%) was also significantly more prevalent among the Brazilian aEPEC than the global aEPEC (Table 5).

**Table 5:** Virulence and antimicrobial resistance genes differentially detected between Brazilian versus Global distributed aEPEC genomes.

Gene	Gene Cluster	No. (%) of aEPEC genomes		*P* value^a^
		Brazil (*n* = 106)	Global (*n* = 221)	
Gene clusters more frequent among Brazilian aEPEC (*P*<0.001)				
***espC***	1024_15_1_31	29 (27.4)	16 (7.2)	<0.0001
	276_12_73_9	28 (26.4)	18 (8.1)	<0.0001
***espT***	148_12_48_29	23 (21.7)	8 (3.6)	<0.0001
	CYEQ01000057.1_8	19 (17.9)	7 (3.2)	<0.0001
***espG2***	128_11_7_153	22 (20.8)	15 (6.8)	0.0004
Gene clusters more frequent among aEPEC from other origins (*P*<0.001)				
***clpV***	401091_31_7	52 (49.1)	154 (69.7)	0.0005
***nleA***	148_12_80_8	39 (36.8)	132 (59.7)	0.0002
***espW***	103338_65_6	8 (7.5)	56 (25.3)	0.0003
***iha***	100329_168_1	7 (6.6)	50 (22.6)	0.0006
***ospB***	C341_10_332_45	26 (24.5)	98 (44.3)	0.0009
***sul2***	CYGQ01000108.1_5	16 (15.1)	90 (40.7)	<0.0001
***bla***_***TEM-1***_	100329_76_20	18 (17.0)	86 (38.9)	0.0001
***aph(6)-Id***	100329_76_18	12 (11.3)	86 (38.9)	<0.0001
***tetA***	DEC11A_c104_2	8 (7.5)	53 (24.0)	0.0006

^a^Differences in the prevalence of gene clusters among the two groups of aEPEC studied (Brazilian aEPEC versus Global aEPEC) were tested using the Chi-square test with Yates correlation.

Conversely, 446 genes were statistically less frequent (*P*<0.001) among the Brazilian aEPEC when compared to the global aEPEC from outside Brazil, including genes encoding a type VI secretion ATPase (49.1% vs 69.7%), the T3SS-effectors *nleA* (36.8% vs 59.7%), *ospB* (24.5% vs 44.3%) and *espW* (7.5% vs 25.3%), and the IrgA homolog adhesin *iha* (6.6% vs 22.6%) (Table 5). Curiously, genes encoding proteins that mediate antimicrobial resistance were also statistically less frequent (*P*<0.001) among the Brazilian aEPEC genomes compared to the aEPEC genomes from outside Brazil, as follows: *bla*_*TEM*-1_ (17.0% vs 38.9%) encoding a TEM-1 beta-lactamase, *sul2* (15.1% vs 40.7%) encoding a sulfonamide resistant dihydropteroate synthase, *aph(6)-Id* (11.3% vs 38.9%) encoding an aminoglycoside phosphotransferase, and *tetA* (7.5% vs 24.0%) encoding an antibiotic efflux pump (Table 5). Other genes that were differentially detected among Brazilian and global aEPEC have predicted functions associated with central metabolism and transcriptional regulation, as well as numerous hypothetical proteins (S5 Table).

### Virulence genes identified among the Brazilian aEPEC that exhibit lineage specific distributions

We also investigated the differences in gene content between Brazilian and global aEPEC isolates identified in the same phylogenomic lineage. Statistically different prevalences (*P*<0.001) were observed in five phylogenomic lineages (EHEC1, EHEC2, EPEC7, EPEC10 and EPEC11) between gene content of the groups of aEPEC genomes analyzed (S6 Table). These included the *toxB* gene, which was first characterized in the pO157 plasmid of EHEC O157:H7 [51]. Interestingly, *toxB* was detected in four Brazilian aEPEC (50.0%, 4/8) that were serotype O26:H11 and identified in the EHEC2 lineage, while the EHEC2 isolates analyzed from outside Brazil lack this gene (S6 Table). In addition, the autotransporter-encoding gene *sepA* (85.7% vs 5.9%) and the T3SS-effector gene *espK* (71.4% vs 0.0%) were statistically more prevalent (*P*<0.001) among the EPEC7 Brazilian aEPEC genomes than the EPEC7 aEPEC from outside Brazil (S6 Table).

We also observed that genes from the PAI OI-122 were statistically (*P*<0.001) different among Brazilian aEPEC compared to global aEPEC in two phylogenomic lineages: EPEC7 and EPEC10 (S6 Table). The OI-122 is a 23-kb chromosomal PAI that consists of three distinct modules, and has been associated with virulence in EHEC and EPEC [52, 53]. Virulence factor-encoding genes can be found in each one of these three modules, as follows: module 1: *pagC*; module 2: *espL2*, *nleB* and *nleE*; and module 3: *efa1* and *efa2* (in O157:H7 EHEC EDL933), or *efa1*/*lifA* (in tEPEC E2348/69) [35, 52, 54]. All seventeen global aEPEC identified in the EPEC7 lineage contained genes of module 2 and module 3, while the Brazilian aEPEC isolates lack these genes (S6 Table). In contrast, the *pagC* (module 1) and *efa1*/*lifA* (module 3) genes were detected in seven (36.8%, 7/19) Brazilian aEPEC identified in the EPEC10 lineage (serotype O2:H16), and in none of the global aEPEC (S6 Table).

### NleF-encoding gene is associated with aEPEC diarrheal disease in Brazil

**Fig 2:**
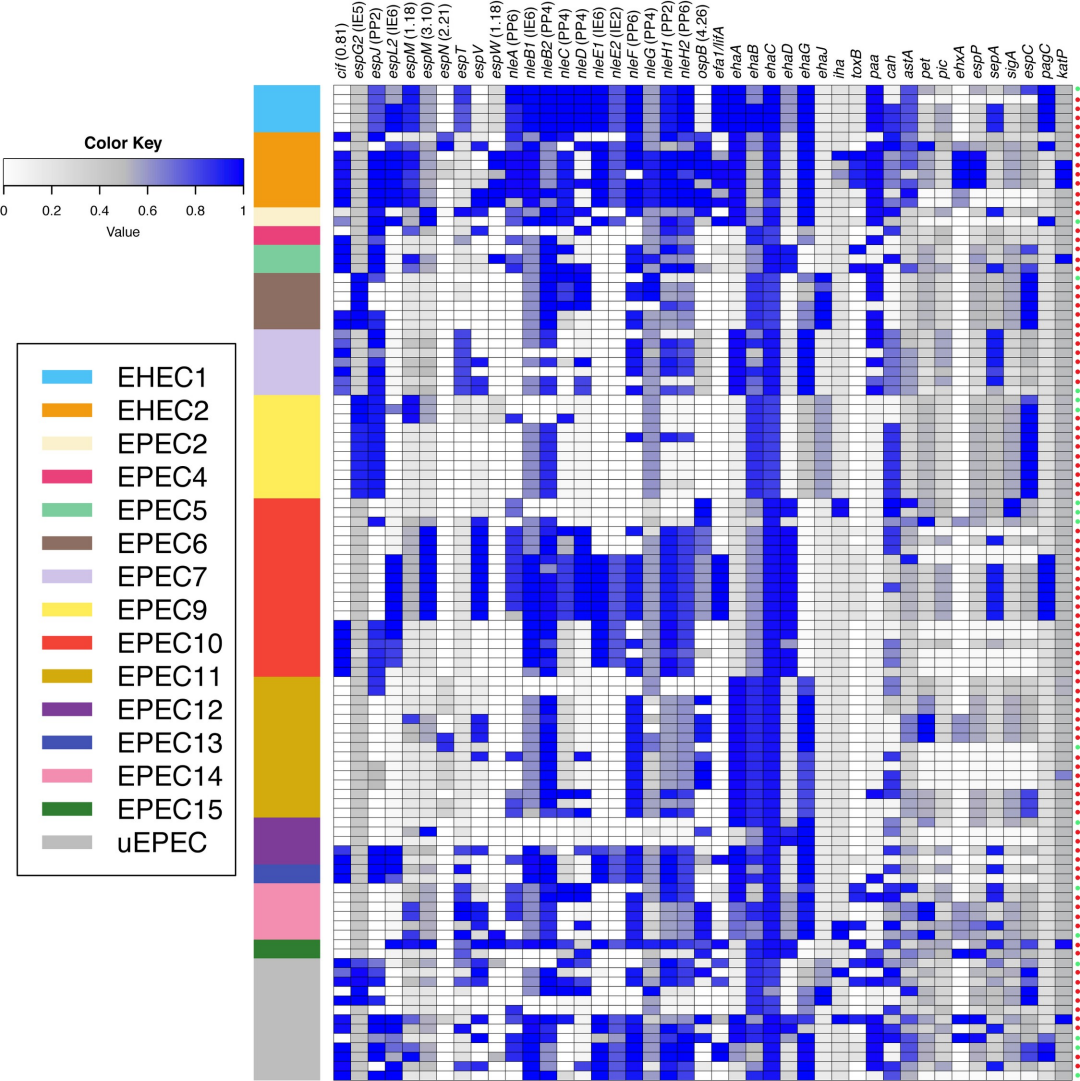
*In silico* detection of known EPEC virulence factor-encoding genes in the 106 Brazilian aEPEC genomes studied. The virulence factor-encoding genes were detected in each of the 106 Brazilian aEPEC genomes analyzed in this study using large-scale BLAST score ratio (LS-BSR) analysis. Each row is a different aEPEC genome, grouped by phylogenomic lineage, and each column represents a single gene (listed in the S7 Table). The presence or absence of all predicted protein-encoding genes in the 106 Brazilian aEPEC genomes is indicated by the BSR values represented in the heatmap generated by using the heatmap2 function of gplots v.3.0.1 in R v.3.3.2. Colors of the heat map indicate virulence genes that were detected with significant similarity (blue), with divergent similarity (gray) or were absent (white) in each of the Brazilian aEPEC genomes analyzed. On the far right red circles indicate aEPEC isolates obtained from patients with diarrhea, while green circles indicate aEPEC isolates obtained from healthy subjects.

Since the Brazilian aEPEC isolates studied here were obtained from patients with diarrhea (89 isolates) as well from healthy subjects (17 isolates), we decided to compare if any of the 43 genes encoding virulence factors investigated was associated with their ability to cause illness in the human host. The number of virulence factor-encoding genes detected in aEPEC isolates obtained from patients with diarrhea ranged from 3 to 23, whereas in those from healthy subjects ranged from 5 to 18 (Fig 2). Of importance, the *nleF* gene was significantly more prevalent among aEPEC isolates obtained from diarrheagenic patients than from healthy subjects (69.7% vs 41.2%, *P*<0.05) (S7 Table).

## Discussion

As prior studies have focused on investigating the genomic diversity of EPEC isolates from locations in Europe, Africa, and Asia [22, 23], the genomic diversity of aEPEC from Brazil or other countries in South America remains largely unexplored. Thus, in this study we describe the genomic diversity of a collection of 106 human aEPEC isolates from Brazil. We also considered the genomic diversity of the Brazilian aEPEC compared to a global collection of aEPEC isolates to gain insight into how Brazilian aEPEC may differ in their evolutionary relatedness and virulence gene content compared with aEPEC from outside of Brazil. By examining the prevalence of known EPEC virulence factors, we determined that the T3SS non-LEE encoded effectors *espG2* [55] and *espT* [37, 56], and the autotransporter gene *espC* [50] were statistically more prevalent among aEPEC from Brazil compared to the global aEPEC from outside Brazil. Of particular interest is EspT, which was functionally characterized in aEPEC archetype isolate E110019 from one of the more several diarrheal outbreaks associated with aEPEC as there were 650 individuals in a school and 137 associated household members in Finland that developed diarrhea [49]. EspT is responsible for triggering the formation of lamellipodia and membrane ruffles, through the activation of Rac-1 and Cdc42 [37], thus facilitating the bacterial invasion into non-phagocytic cells [56]. Although the occurrence of invasive aEPEC in the Brazilian settings have already been reported [57], no correlation between this phenotype and the presence of the *espT* gene has been observed to date. EspG2 is also of importance as it is involved in microtubule disruption [55]. EspC, a member of the autotransporter family, is involved in EPEC-mediated cell death and induces both apoptosis and necrosis in epithelial cells [58]. In tEPEC archetype isolate E2348/69 the *espC* and *espG2* (*orf3*) are located in the same PAI, termed integrative element (IE) 5 or EspC PAI [35, 59], which may explain the simultaneous dissemination of these two genes among the Brazilian aEPEC isolates. In addition, we observed that *nleF* was the only virulence factor-encoding gene that demonstrated a statistical association with the isolates from diarrheal disease cases when compared to Brazilian aEPEC isolates from asymptomatic individuals. NleF is a Non-LEE effector, secreted to the host cells by the T3SS, that binds to caspase-4, -8, and -9 inhibiting the catalytic activity of these caspases *in vitro*, and therefore, prevents apoptosis in HeLa and Caco-2 cells [60].

We also observed that the Brazilian aEPEC differ in their gene content at the phylogenomic lineage level, when compared with aEPEC from other geographic origins. In particular, the *toxB* gene of pO157 from O157:H7 EHEC strains, and *efa1*/*lifA* of the PAI OI-122 [54, 61], were more prevalent among Brazilian aEPEC identified in the EHEC2 and EPEC10 phylogenomic lineages, respectively. The proteins encoded by the *toxB* and *efa1*/*lifA* genes are associated with the ability of EHEC strains to adhere to epithelial cells. Of note, the EHEC adherence factors *efa1* (Z4332) and *efa2* (Z4333) genes, located in the third module of the PAI OI-122 from EHEC O157:H7 strains [52], can encode proteins with amino acid sequences that are identical to the amino acids 1 to 433 (99.0% of identity) and 437 to 711 (100.0% identity) of the Efa1 adhesin identified in the EHEC O111:H^-^ [62]. A previous study, using random transposon mutagenesis, demonstrated that the EHEC O157:H7 Sakai strain, harboring a transposon insertion in the *efa1* gene, was significantly less adherent than the wild type strain in assays performed with Caco-2 cells [63]. The nucleotide sequence of the *efa1* gene, from the EHEC O111:H^-^ strain, is 99.0% identical to the *lifA* gene (also termed *efa1*/*lifA*) from the tEPEC E2348/69, that has been demonstrated to inhibit lymphocyte proliferation and lymphokine production [64]. A clinical study, that evaluated stool samples from three distinct children with diarrhea associated with an EHEC isolate with the serotype O157:HNM, demonstrated that EHEC may lose the *stx2* gene during trafficking in the host [65]. This line of evidence suggests that some of the aEPEC isolates in our study, which were genomically related to EHEC lineages and/or harbored virulence genes associated with this pathotype may in fact be EHEC that have lost the Shiga toxin genes during trafficking in the host or environment, or during culture in the clinical laboratory.

Overall, this study demonstrated the aEPEC from Brazil are genomically diverse, with representatives in nearly all of the *E*. *coli* phylogroups and previously described EHEC and EPEC phylogenomic lineages, which is similar to what has been previously described for aEPEC from outside of Brazil. The Brazilian aEPEC genomes described in this study not only provide insight into the genomic diversity of aEPEC in Brazil, but also allowed the description of five novel EPEC phylogenomic lineages. We also identified important differences in gene content, including known virulence associated genes, between Brazilian aEPEC compared to a global collection of aEPEC from outside Brazil. Further investigation of the genomic diversity and prevalence of select virulence genes among additional aEPEC from Brazil as well as other locations in South America may provide insight into the greater prevalence of some of these lineages of aEPEC in association with diarrheal illness in Brazil.

## Supporting information

S1 Data Nucleotide sequence of the gene clusters in fasta format. (FASTA)

S1 Fig World map indicating the countries from which the globally distributed aEPEC collection was obtained. Atypical EPEC from the global collection was obtained from all five continents, mainly from Africa, Asia, and Europe. In parentheses is the number of aEPEC isolates obtained from each country. This world map was constructed using the free-software Q-GIS.
(TIFF)

S1 Table Brazilian atypical EPEC (aEPEC) strains sequenced in the present study. (XLSX)

S2 Table Global collection of atypical enteropathogenic *Escherichia coli* (aEPEC) strains from outside Brazil. (XLSX)

S3 Table *Escherichia coli* and *Shigella* reference genomes included in the phylogenomic analysis. (XLSX)

S4 Table LS-BSR identification of gene clusters that are unique in the five novel phylogenomic lineage identified in this study. (XLSX)

S5 Table Gene clusters differentially detected in the Brazilian aEPEC genomes in comparison with aEPEC from outside Brazil. (XLSX)

S6 Table Gene clusters differentially detected among aEPEC from Brazil and from other origins by phylogenomic lineage. (XLSX)

S7 Table Occurrence of virulence factor-encoding genes in aEPEC obtained from diarrheagenic patients and healthy subjects in Brazil. (XLSX)
