# Supplementary figures and images for: Comparative genomic analysis provides insight into the phylogeny and virulence of atypical enteropathogenic Escherichia coli strains from Brazil
Source: PLoS Negl Trop Dis. 2020 Jun 1;14(6):e0008373. doi: 10.1371/journal.pntd.0008373 (PMC7289442; doi:10.1371/journal.pntd.0008373)

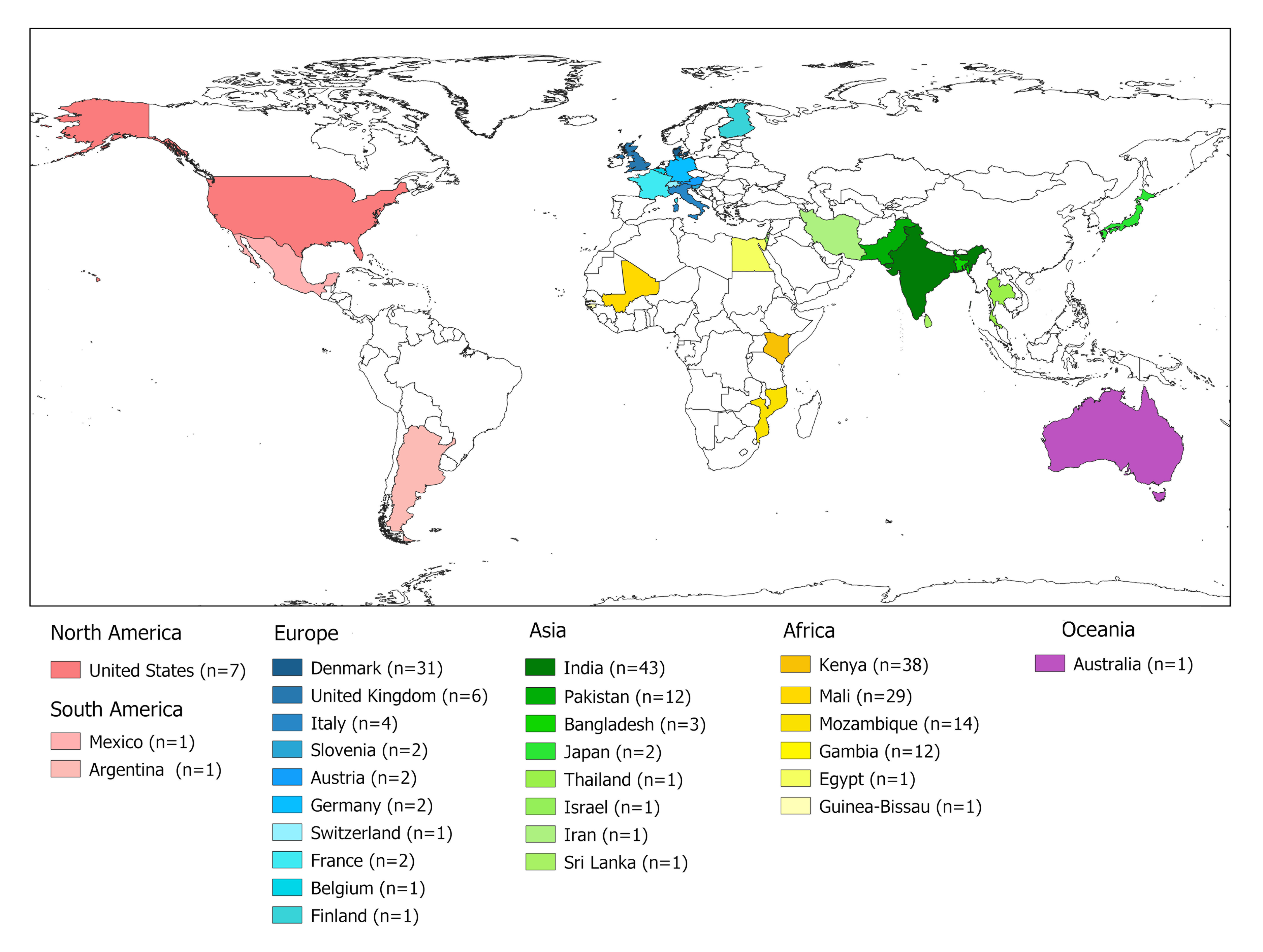

Supplement: S1 Fig — Atypical EPEC from the global collection was obtained from all five continents, mainly from Africa, Asia, and Europe. In parentheses is the number of aEPEC isolates obtained from each country. This world map was constructed using the free-software Q-GIS. (TIFF) [file pntd.0008373.s002.tiff]
